# Enhancing electron transfer of a semiconducting polymer for type I photodynamic and photothermal synergistic therapy

**DOI:** 10.3389/fbioe.2022.1004921

**Published:** 2022-09-19

**Authors:** Cao Cui, Xuehua Su, Yongchun Guo, Jun Zhu, Zimeng Chen, Wei Qin, Yihang Guo, Wenming Tao

**Affiliations:** Xiangyang Central Hospital, Affiliated Hospital of Hubei University of Arts and Science, Xiangyang, Hubei, China

**Keywords:** PDPP, human cervical cancer, nanoparticles, type I PDT, PTT

## Abstract

Tumor hypoxia is responsible for the reduced therapeutic efficacy of type II photodynamic therapy (PDT) because of the dependence of cellular oxygen during ^1^O_2_ generation. Type I PDT may be a better strategy to overcome the disadvantages of hypoxia for enhanced theranostics. Herein, a new semiconducting polymer PDPP was synthesized and encapsulated with hydrophilic PEG-PDPA to enhance the electron transfer for type I PDT. PDPP NPs show a high superoxide radical generation ability with DHR123 as a probe. *In vitro* MTT assay indicates PDPP NPs with considerably high phototoxicity on human cervical cancer cells (HeLa) with a low half-maximal inhibitory concentration (IC_50_) of 6.1 μg/ml. Furthermore, an *in vivo* study demonstrates that PDPP NPs can lead to complete tumor suppression with the help of laser, compared with the control and dark groups. The biosafety is confirmed by the H&E analysis of the normal tissues (the heart, liver, spleen, lungs, and kidney). The results provide a strategy to design nanosystems for type I PDT and PTT synergistic therapy.

## Introduction

Phototherapy, including photodynamic and photothermal therapy, holds tremendous potential for cancer treatment because it utilizes the photogeneration of cytotoxic reactive oxygen species (ROS) or heat, respectively, to induce cell apoptosis and further leads to tumor suppression (Zhou et al., 2016; Xu et al., 2017; Huang et al., 2018; Li et al., 2019a; Liu et al., 2019a; Liu et al., 2019b; Li et al., 2020a; Li et al., 2020b; Yao et al., 2020; Zhang et al., 2020b; Tahir et al., 2021; Wang et al., 2021). Continuous irradiation, however, inevitably leads to tumor hypoxia in the photoinduced ROS generation, especially the oxygen-dependent type II process (Zou et al., 2020; Zou et al., 2021a). This will result in reduced oxygen supply and decreased therapeutic efficacy.

Type I PDT is based on the electron/hydrogen transfer to generate cytotoxic superoxide (O_2_^−^) and hydroxyl radicals (OH) (Fan et al., 2016; Zhang et al., 2020a). OH is derived from intercellular hydrogen peroxide or water, promising a reduced dependence upon cellular oxygen. Type I PDT, therefore, may be a better strategy to fight against hypoxic tumors. In recent years, considerable attention has been attached to semiconducting polymer-based theranostics, owing to their unique properties, such as near-infrared absorbance, excellent photothermal conversion efficiency, and ROS generation ability (Tang et al., 2018; Li et al., 2019b; Li et al., 2019c; Shen et al., 2019; Yang and Chen, 2019; Deng et al., 2020; Yang et al., 2020; Zhang et al., 2020a; Zhang et al., 2021). For example, Zou et al. (2020) designed a heavy atom-free compound for efficient singlet oxygen generation and continuous PDT. Semiconducting polymers show great potential in molecular imaging and theranostics.

**SCHEME 1:**
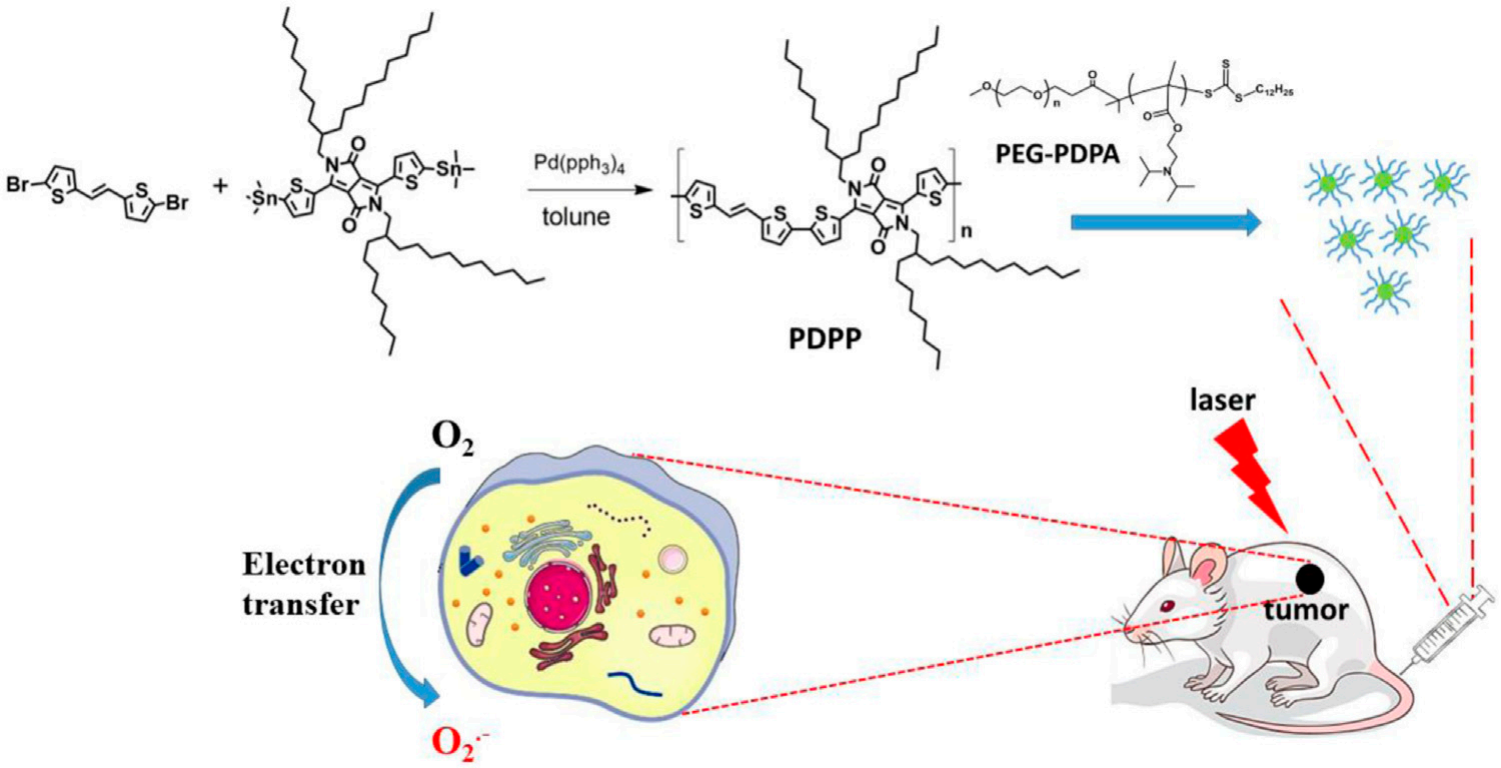
Synthesis route of PDPP and NPs for phototheranostics.

In this work, a semiconducting polymer poly(*E*)-3-(5-methylthiophen-2-yl)-6-(5′-(2-(5-methylthiophen-2-yl)vinyl)-[2,2′-bithiophen]-5-yl)-2,5-bis(2-octyldodecyl)pyrrolo[3,4-c]pyrrole-1,4(2H,5H)-dione (denoted as PDPP NPs) was prepared by treating (*E*)-1,2-bis(5-bromothiophen-2-yl)ethene and 2,5-bis(2-octyldodecyl)-3,6-bis(5-(trimethylstannyl)thiophen-2-yl)pyrrolo[3,4-c]pyrrole-1,4(2H,5H)-dione through a Stille polymerization reaction (Scheme 1). PDPP was characterized by NMR. Then, PEG-PDPA with a strong electron transfer ability was used to encapsulate PDPP by nanoprecipitation to prepare nanoparticles (NPs) with good water dispersion. Such NPs exhibit a spherical morphology with an average diameter of 55 nm. The superoxide radical generation of PDPP NPs was enhanced dramatically, as indicated by the fluorescence enhancement of DHR123 with laser irradiation. In addition, the photothermal conversion efficiency of PDPP NPs is as high as 32.3%, promising a synergistic effect against cancer. The cytotoxicity determined by the MTT assay *in vitro* indicates that such NPs have a low half-maximal inhibitory concentration (IC_50_) of 6.1 μg/ml on human cervical cells (HeLa) by laser irradiation. With the help of laser, PDPP NPs are capable of inhibiting tumor proliferation without adverse effects on normal tissues, including the heart, spleen, liver, kidney, and lung. These results suggest that PDPP NPs show great potential for cancer phototheranostics.

## Experimental section

### Materials and apparatus

^1^H NMR spectra were observed using a Bruker DRX NMR spectrometer in CDCl_3_ (*δ* = 7.26 ppm) at 298 K as the internal standard. UV-vis and fluorescence spectra were measured using a Shimadzu spectrophotometer from Japan (UV-3600) and a FS970 spectrometer (Japan), respectively. TEM of the nanoparticles was measured on equipment (JEOL JEM-2100). Dynamic light scattering (DLS) of PDPP NPs was tested using a particle size analyzer (90 Plus, Brookhaven Instruments, United States).

### Synthesis and characterization of PDPP

A mixture of (*E*)-1,2-bis(5-bromothiophen-2-yl)ethene (171.0 mg, 1.0 mmol) and 2,5-bis(2-octyldodecyl)-3,6-bis(5-(trimethylstannyl)thiophen-2-yl)pyrrolo[3,4-c]pyrrole-1,4(2H,5H)-dione (1171.0 mg, 1 mmol), Pd(PPh_3_)_4_ (115.6 mg, 1 mmol) was dissolved in 5 ml of toluene. Then, N_2_ was bubbled to drive off the possible oxygen and water in the system. The mixture was heated at 110°C under the protection of N_2_ for 12 h. After cooling at room temperature, the mixture was poured into cold methanol (200 ml), and the crude product was filtered. Then, the crude product was dissolved in tetrahydrofuran and precipitated in methanol three times. The ^1^HNMR spectrum is shown in Supplementary Figure S1 ^1^HNMR: δ H.

### Cell culture and MTT assay

At 37°C, human cervical cancer cells (HeLa) were cultured in a medium consisting of 12% fetal bovine serum (FBS) in DMEM (Gibco) under an atmosphere of 5% CO_2_. PDPP NPs with different concentrations were co-cultured with HeLa cells in the 96-well plate. For the group with irradiation, each well was irradiated with a 730-nm laser for 8 min. In contrast, the wells in the control and the one without irradiation were not irradiated. Relative cell viability was determined by the absorbance of MTT [3-(4,5-dimethylthiazol-2-yl)-2,5-diphenyltetrazolium bromide]. MTT in PBS (5 mg/ml) was added to the well (20 μl), followed by incubation for 4 h. After that, the medium was discarded, and DMSO (200 μl) was added. The absorbance of each well was recorded on a Bio-Tek microplate reader. Cell viability was then calculated according to the equation:

Cell viability (%) = mean absorbance of the group incubated with PDPP NPs/mean absorbance of the group.

### Cellular uptake, fluorescence imaging of cellular ROS, and sub-organelle co-localization

HeLa cells were cultured with PDPP NPs (3 ml) in a confocal dish for 24 h. Then, the medium was discarded, and the cells were washed with PBS (1 ml) three times. Polyoxymethylene (1 ml) was added for 25 min to fix the cells. Then, polyoxymethylene was discarded, and the cells were also washed with PBS three times (1 ml). The cells were further co-cultivated with dihydrorhodamine 123 (DHR123, 10 μmol) for 5 min. A 730-nm laser was then irradiated onto the sample for 3 min (0.5 W/cm^2^). For the cellular uptake, the cells were excited at 633 nm, and the fluorescence was collected from 650 to 750 nm. For *in vitro* superoxide radical generation, the cells were excited with a 488-nm laser, and fluorescence was collected from 495 to 550 nm to show the ROS generation.

### Photothermal imaging guided phototherapy

All animal studies were performed following guidelines approved by the Ethics Committee of the Xiangyang Central Hospital, Affiliated Hospital of Hubei University of Arts and Science. A total of 15 nude mice were purchased and then inoculated with HeLa cells. Three mice have been chosen to perform *in vivo* fluorescence imaging. The fluorescence image was captured first, and then, the three mice were intravenously injected with PDPP NPs, and the fluorescence imaging pictures were also captured at different time points. A total of 12 nude mice were divided into three groups at random when the tumor volume reached about 80 mm^3^. For the dark and illumination groups, the mice were intravenously injected with PDPP NPs (200 μg/ml, 100 μl). After 12 h, the tumors of the PBS + laser and PDDP + laser groups were irradiated by a 730-nm laser (1 W/cm^2^) for 8 min, while the mice treated with PDPP NPs only were not irradiated. These nude mice were then sacrificed for histological analysis.

## Results and discussion

### Synthesis and generation characterization of PDPP NPs

**FIGURE 1:**
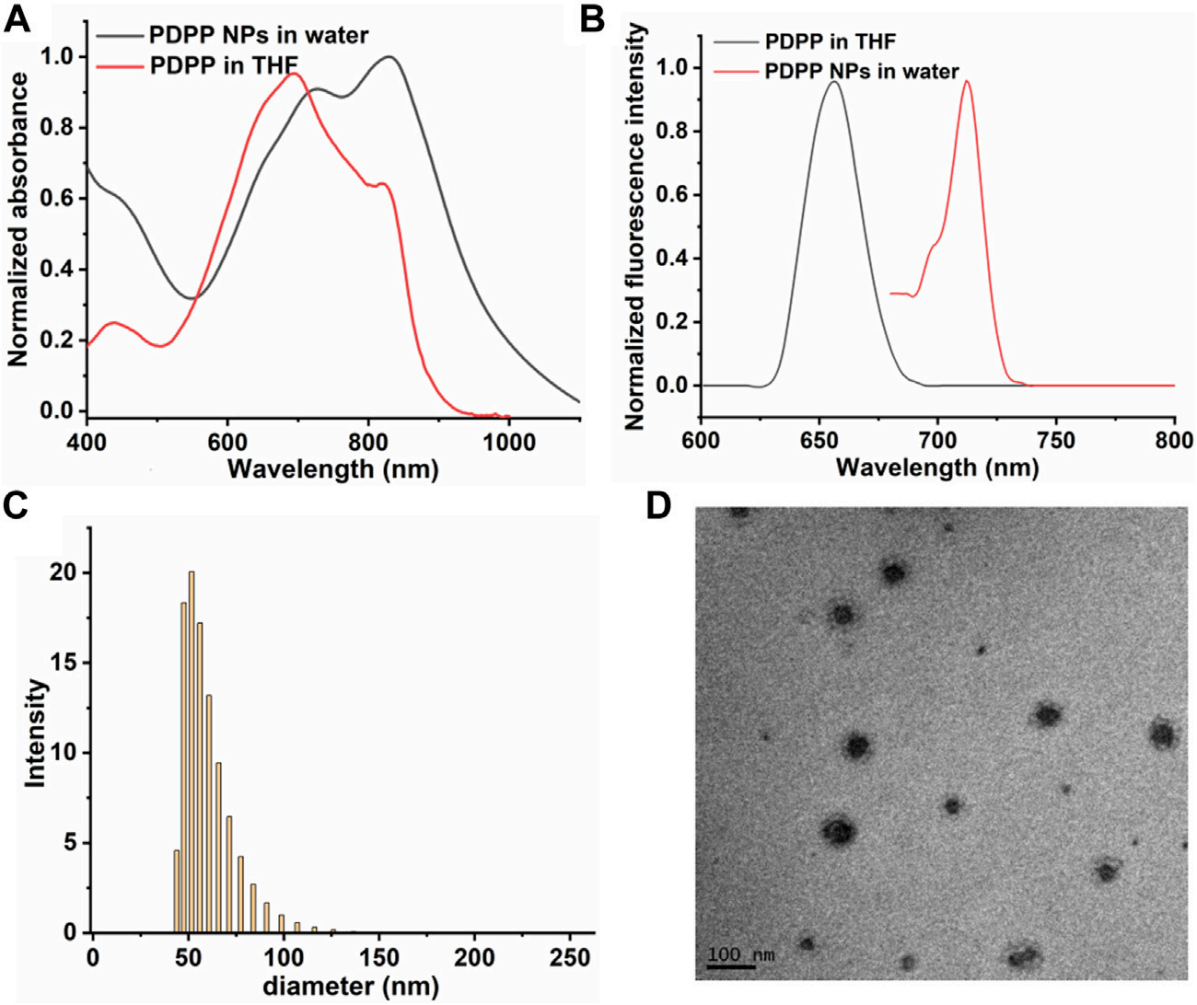
Normalized **(A)** absorbance spectra of PDPP NPs. **(B)** Fluorescence spectra of PDPP in THF and NPs in water. **(C)** TEM of PDPP NPs. **(D)** DLS of PDPP NPs in water.

The photophysical properties of PDPP NPs were characterized by UV-vis spectroscopy and fluorescence emission. PDPP in THF shows absorption peaks at 691 and 815 nm, while that of the NPs in water is shifted to 712 and 829 nm, respectively, indicating their potential to respond to near-infrared (NIR) light (Figures 1A,B). A large Stokes shift was observed for the maximum absorbance of PDPP NPs in water (616 and 719 nm), which is ascribed to the aggregation of PDPP NPs in the aqueous solution. In addition, both transmission electron microscopy (TEM) and dynamic light scattering (DLS) Figure 1C show that such NPs with uniform size (mean diameter ∼58 nm) exhibit spherical morphology (Figure 1D).

### Superoxide radical generation and photothermal conversion

**FIGURE 2:**
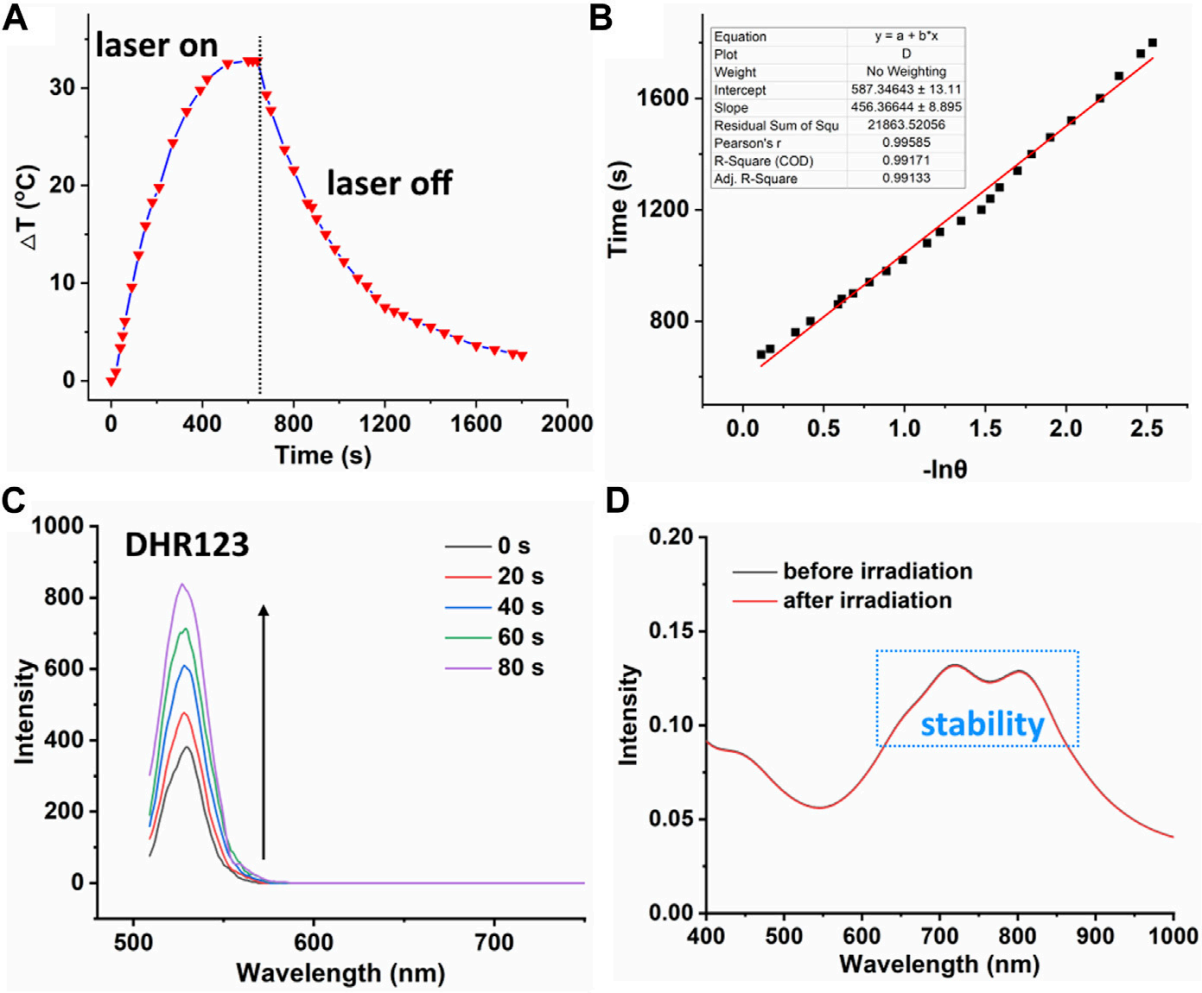
**(A)** Temperature elevation and decrease curve of PDPP NPs (730 nm, 500 W/cm^2^, 10 min). **(B)** Linear fitting of −lnθ versus time. **(C)** Fluorescence intensity of DHR123 with laser irradiation (730 nm, 50 W/cm^2^). **(D)** Absorbance spectra of PDPP NPs before and after irradiation.

On the one hand, high photothermal conversion efficiency (PCE) promises high phototherapeutic efficacy (Turan et al., 2016; Zhu et al., 2019; Zou et al., 2021b). The heating curve of PDPP NPs in distilled water with irradiation or the cooling curve without irradiation was recorded (Figure 2A). The temperature elevation of 32.5°C was observed with laser irradiation in the presence of PDPP, and the photothermal conversion efficiency is as high as 32.3% (Figure 2B). On the other hand, the high ROS generation ability promises high phototoxicity. The superoxide radical generation of PDPP NPs was determined by recording the fluorescence intensity of DHR123 with laser irradiation. With the help of the laser, the fluorescence intensity of DHR123 is three times higher than that of the original one, while that of DHR123 without irradiation is almost negligible (Figure 2C; Supplementary Figure S2). It is worthy of being noted that PDPP NPs are heavy-atom-free, and this may reduce the potential dark toxicity by themselves. The absorbance before and after irradiation can be ignored, indicating the excellent photostability of such NPs (Figure 2D).

### *In vitro* cellular uptake, O_2_^−^ generation, and MTT assay

**FIGURE 3:**
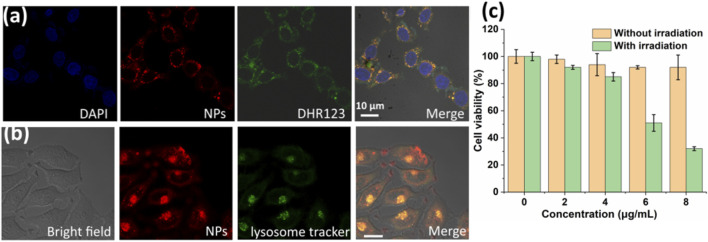
**(A)** *In vitro* cellular uptake and superoxide radical generation with DHR123 as a probe (730 nm, 100 W/cm^2^). **(B)** Lysosome co-localization with PDPP NPs. **(C)** Cell viability of HeLa cells in the presence of PDPP NPs at different concentrations (0, 2, 4, 6, and 8 μg/ml) (730 nm, 500 W/cm^2^, 8 min).

Owing to the high O_2_^−^ generation ability and photothermal conversion efficiency, the therapeutic efficacy of PDPP NPs was investigated *in vitro*. First, the cellular uptake and O_2_^−^generation were determined by confocal laser scanning microscopy (CLSM). PDPP NPs can be uptaken by HeLa cells after incubation for 24 h (Figure 3A). Strong O_2_^−^generation could be observed due to the strong green fluorescence (Figure 3A). Such NPs can localize in the lysosomes, as suggested by the co-localization experiment (Figure 3B). Then, the dark phototoxicity was determined by the MTT assay. For the dark group, the cell viability was independent upon the concentration, indicating the low dark toxicity of PDPP NPs (Figure 3C). In comparison, the cell viability in the group with irradiation showed concentration-dependent death, and the half maximal inhibitory concentration of PDPP NPs is 6.1 μg/ml (Figure 3C). The results demonstrated that PDPP NPs show potential for PDT/PTT synergistic therapy.

### *In vivo* type I photodynamic and photothermal therapies

**FIGURE 4:**
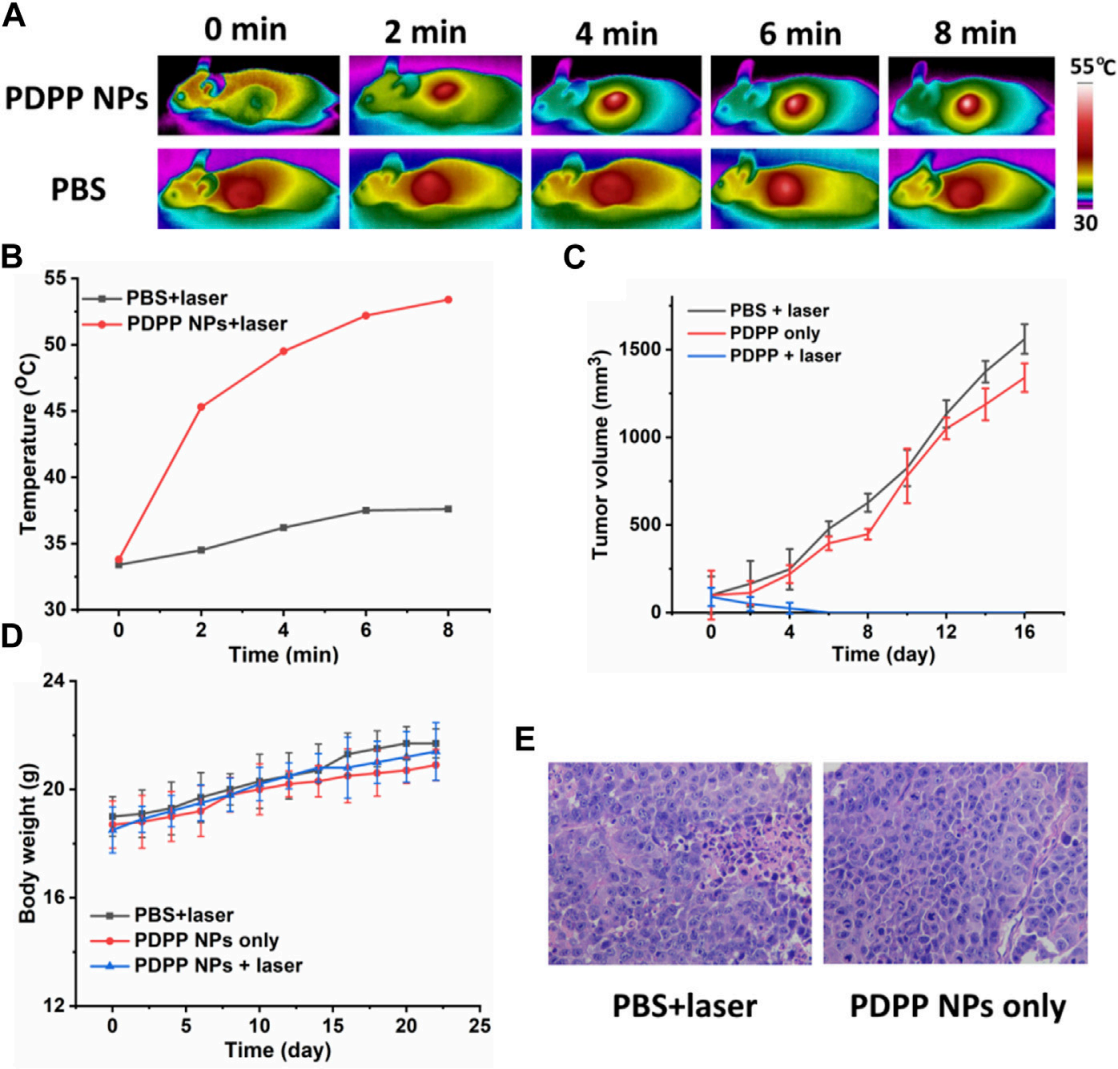
**(A)** Time-dependent photothermal imaging of tumor with irradiation (730 nm, 500 W/cm^2^, 8 min). **(B)** Quantification of tumor temperature at different time intervals. **(C)** Tumor volume of the mice in PBS + laser, PDPP NP-only, and PDPP NPs + laser groups (730 nm, 500 W/cm^2^, 8 min). **(D)** Body weight change. **(E)** H&E-stained pictures of the tumors in the PBS + laser and PDPP NP-only groups.

Inspired by the excellent phototherapeutic efficacy of PDPP NPs *in vitro*, further *in vivo* study has been investigated. First of all, time-dependent photothermal imaging of the mice with laser irradiation was recorded (Figure 4A). A significant temperature elevation of approximately 20°C was observed for the tumor with laser irradiation for 8 min, while that of the control group was only 4.0°C (Figures 4A,B), indicating the excellent photothermal efficacy of PDPP NPs *in vivo*.

Then, the tumor volume and body weight were recorded every 2 days. The tumor volume of the mice administered with PDPP NPs was almost the same as that of the PBS + laser group, demonstrating the low dark toxicity of PDPP NPs (Figure 4C). However, the tumor in the PDPP NPs + laser group eventually disappeared after being treated three times, which suggests the distinct therapeutic efficacy of such NPs. All the mice tended to gain more weight in all the three groups, regardless of irradiation or not (Figure 4D). The H&E-stained pictures of the tumors in the PBS + laser and PDPP NP-only groups are observed in a good manner, indicating the excellent biocompatibility of the NPs (Figure 4E) (Zhen et al., 2017; Chen et al., 2019; Li et al., 2019d; Ma et al., 2019).

**FIGURE 5:**
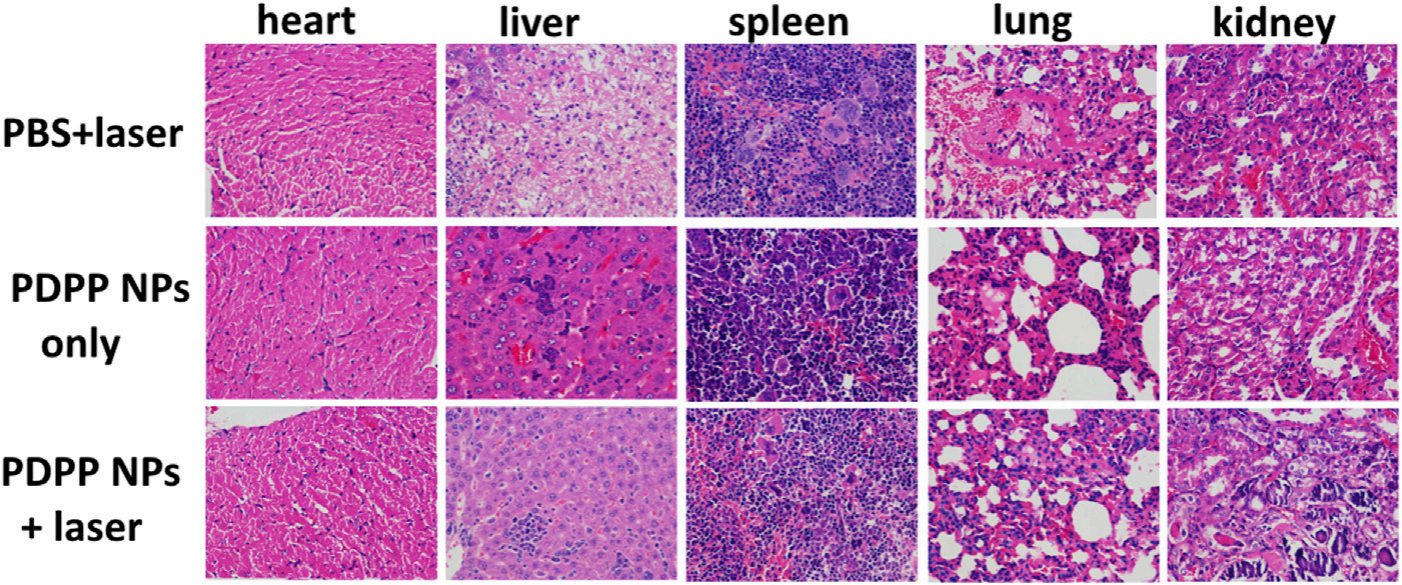
H&E-stained pictures of the normal organs (the heart, liver, spleen, lung, and kidney) in the PBS + laser, PDPP NP-only, and PDPP NPs + laser groups.

All the mice were sacrificed, and the normal organs were collected for the H&E study, including the heart, liver, spleen, lung, and kidney. The morphology of the nucleus was observed in a good manner, and no obvious difference was observed in H&E-stained pictures (Figure 5). All the results demonstrated that PDPP NPs exhibit strong anti-tumor activity and low side effects *in vivo*.

## Conclusion

In summary, a semiconducting polymer PDPP was encapsulated by PEG-PDPA with high electron transfer ability, leading to efficient superoxide radical generation for type I PDT. The as-prepared PDPP NPs also exhibit excellent photothermal conversion efficiency (32.3%). *In vitro* MTT assay shows that PDPP NPs show low dark toxicity but high phototoxicity with a low IC_50_ of 6.1 μg/ml. *In vivo* photothermal imaging suggests that such NPs can suppress tumor growth at a low dose but cause no side effects to normal tissues. These results provide some insights into the design of semiconducting polymer photosensitizers with high phototoxicity, low dark toxicity, and good biocompatibility for photothermal and type I photodynamic therapy.

## Data Availability

The original contributions presented in the study are included in the article/Supplementary Materials; further inquiries can be directed to the corresponding authors.
