# Supplementary material for: Enhancing electron transfer of a semiconducting polymer for type I photodynamic and photothermal synergistic therapy
Source: Front Bioeng Biotechnol. 2022 Sep 19;10:1004921. doi: 10.3389/fbioe.2022.1004921 (PMC9527290; doi:10.3389/fbioe.2022.1004921)
Supplement: Supplementary file 1 [file DataSheet1.docx]

**Enhancing electron transfer of a semiconducting polymer for type I photodynamic and photothermal synergistic therapy**

Cao Cui, Xuehua Su, Yongchun Guo, Jun Zhu, Zimeng Chen, Wei Qin, Yihang Guo*, Wenming Tao*

*Xiangyang Central Hospital, Affiliated Hospital of Hubei University of Arts and Science, Hubei 441021, China. Email:* [*guoyihang87@126.com*](mailto:guoyihang87@126.com)*,* [*wenmingtao66@sina.com*](mailto:wenmingtao66@sina.com)*.*

**Preparation of PDPP nanoparticles**

The nanoparticles of PDPP were prepared by nano-precipitation with PEG-PDPA. PEG-PDPA (10 mg) was dissolved in distilled water with ultrasound. And then PDPP (2 mg) was dissolved in tetrahydrofuran (THF, 1 mL). Then 200 μL of such solution was injected into PBS with ultrasound at room temperature. After the mixture was stirred for 10 min, THF was removed by purging nitrogen. The product was then frozen and dried for further use.

**Detection of superoxide radical generation**

The superoxide radical generation of PDPP NPs in aqueous solution was investigated using DHR123 as a probe. A mixture of PDPP NPs (1 μM) and DHR123 (10 μM) in water was irradiated and the fluorescence was recorded after different periods of time (0, 20, 40, 60 and 80 s).

**Photothermal conversion efficiency of PDPP NPs**

PDPP NPs in water was irradiated by laser and then cooled to room temperature. The temperature was recorded by an infrared camera. The photothermal conversion efficiency was calculated according to equation (1-6).

$$\eta=\frac{hs\left( T_{max}-T_{amb} \right)-Q_{Dis}}{I\left( 1-{10}^{-A660} \right)} (1)$$

$$\theta=\frac{T-T_{amb}}{T_{Max}-T_{amb}} \left( 2 \right)$$

$$dt=-\tau s\frac{d\theta}{\theta} \left( 3 \right)$$

$$\tau_{s}=\frac{\sum_{i} m_{i}C_{p,i}}{hs} \left( 4 \right)$$

$$t=-\tau s\ln(\theta) (5)$$

where *h* is the heat transfer coefficient, *S* is the surface area of the container. The *T*_max_ is the highest temperature of PDPP NPs in water at the maximum steady-state temperature, *I* is the laser power density (0.5 W/cm^2^), *A_660_* is the absorbance of the PDPP NPs at 660 nm and *Q_Dis_* is the heat associated with light absorption by the solvent. The variable *τ_s_* is the sample-system time constant, and m_i_ and *C_i_* are the mass and heat capacity of the deionized water (4.2× 10^3^ J/kg^-1^/^o^C), respectively.


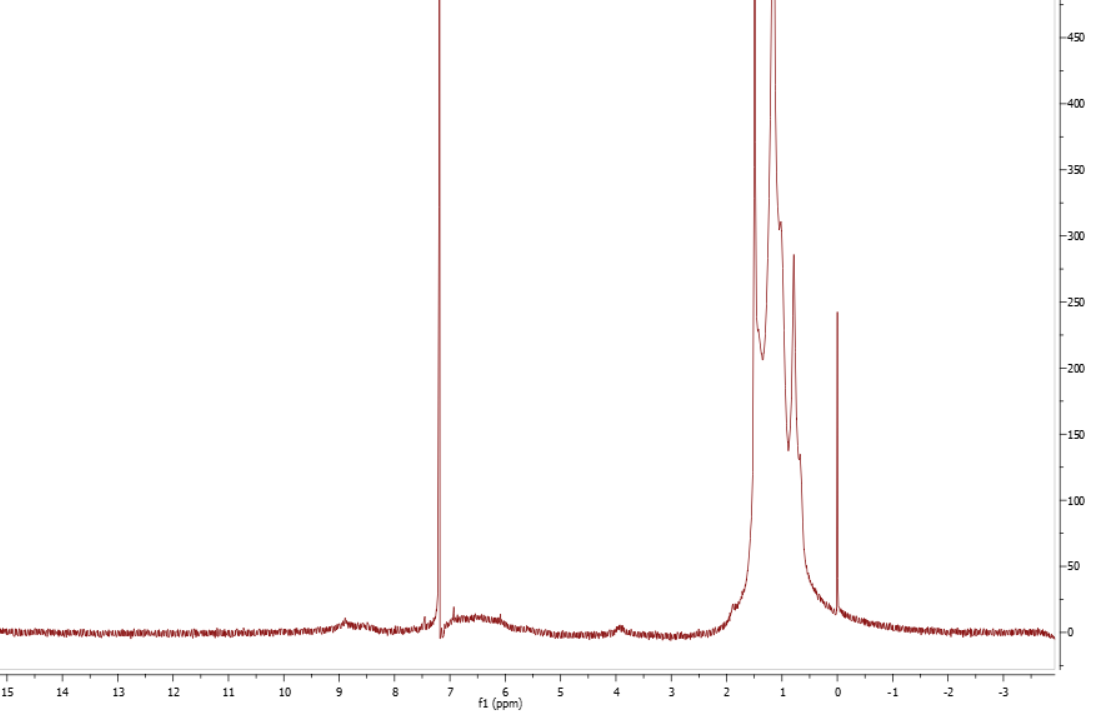


Figure S1 ^1^HNMR of PDPP in CDCl_3._


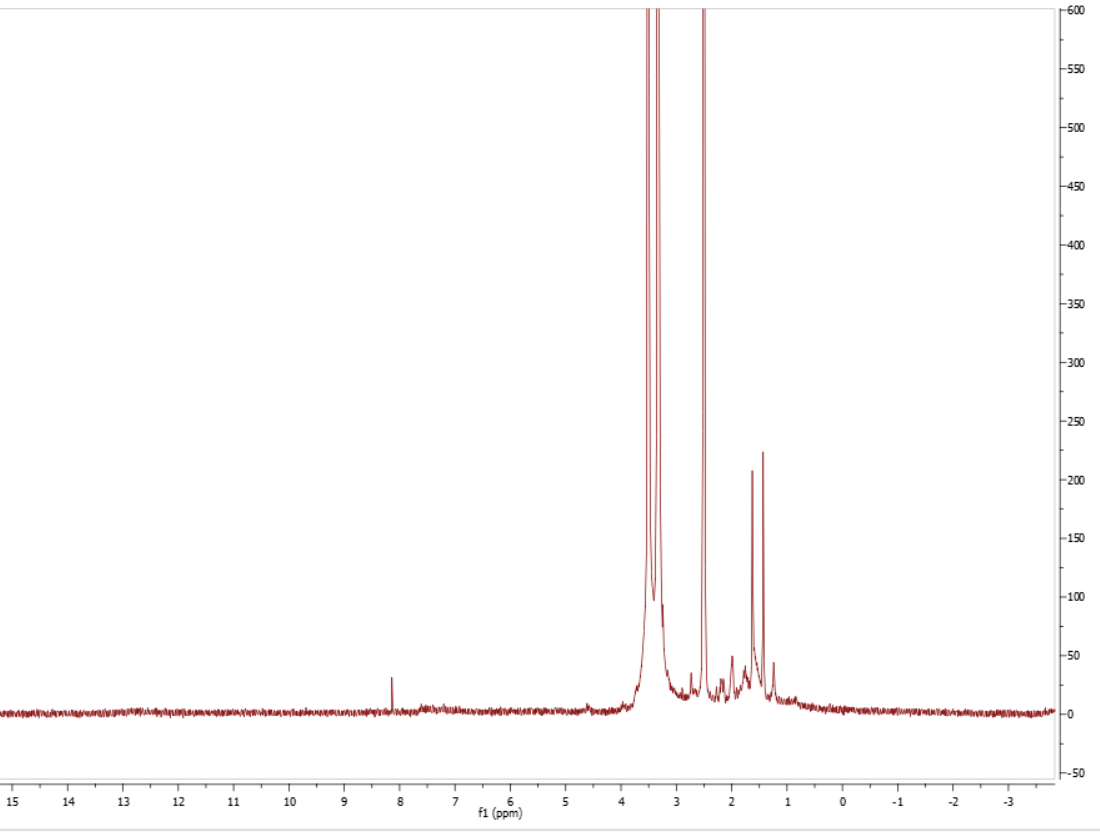


Figure S2 ^1^HNMR of PEG-PDPA in DMSO-d6. _._

*
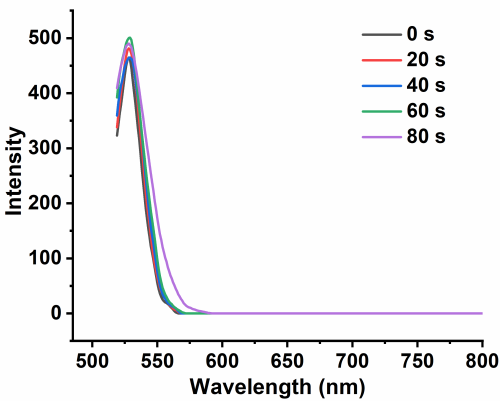
*

Figure S3 Fluorescence intensity of DHR123 with laser irradiation (50 mW/cm^2^).
